# Leukocyte Telomere Length Is Related to Brain Parenchymal Fraction and Attention/Speed in the Elderly: Results of the Austrian Stroke Prevention Study

**DOI:** 10.3389/fpsyt.2020.00100

**Published:** 2020-02-28

**Authors:** Piyush Gampawar, Reinhold Schmidt, Helena Schmidt

**Affiliations:** ^1^Research Unit-Genetic Epidemiology, Gottfried Schatz Research Centre for Cell Signalling, Metabolism and Aging, Molecular Biology and Biochemistry, Medical University Graz, Graz, Austria; ^2^Department of Neurology, Clinical Division of Neurogeriatrics, Medical University Graz, Graz, Austria

**Keywords:** telomeres, leukocyte telomere length, brain aging, cognition, brain parenchymal fraction, white matter hyperintensities, attention/speed

## Abstract

There are controversial results if leukocyte telomere length (LTL) is related to structural brain changes and cognitive decline in aging. Here, we investigated the association between LTL and 1) global MRI correlates of brain aging such as brain parenchymal fraction (BPF) and white matter hyperintensities (WMH) load and Fazekas score as well as 2) global (g-factor) and domain-specific cognition such as attention/speed, conceptualization, memory, and visuopractical skills. In total, 909 participants of the Austrian Stroke Prevention Study with LTL, MRI, and cognitive tests were included. There were 388 (42.7%) men, and the mean age was 65.9 years. Longer LTL was significantly associated with larger BPF (β = 0.43, p < 0.001), larger WMH load (β = 0.03, p = 0.04), and score (β = 0.05, p = 0.04) after adjusting for age, sex, vascular risk factors, and ApoE4 carrier status. The effect on BPF was more significant in the subgroups of women (β = 0.51, p = 0.001), age >65 years (β = 0.58, p = 0.002), BMI ≥ 25 (β = 0.40, p = 0.004), education ≤10 years (β = 0.42, p = 0.002), hypertensives (β = 0.51, p = 0.001), cardiovascular disease (CVD) (β = 0.58, p = 0.005), non-diabetics (β = 0.42, p < 0.001), and Apoe4 non-carriers (β = 0.49, p < 0.001). The effect on WMH was significant within the hypertensives (load: β = 0.04, p = 0.02), non-diabetics (load:β = 0.03, p = 0.01; score: β = 0.06, p = 0.02), in those with education ≤10 years (load: β = 0.03, p = 0.04; score: β = 0.07, p = 0.02), in ApoE4 non-carriers (load: β = 0.03, p = 0.02; score: β = 0.07, p = 0.01) and in subjects without CVD (score: β = 0.06, p = 0.05). We only observed a significant association between LTL and the cognitive domain of attention/speed, which was confined to the subgroups of BMI ≥ 25 (β = 0.04, p = 0.05) and education ≤10 years (β = 0.04, p = 0.05). The effect of LTL on attention/speed was partly mediated in both subgroups by BPF (β = 0.02, 95% CI = 0.01:0.03) when tested by bootstrapping. Our results support a strong protective role of longer LTL on global brain volume which in turn may contribute to better cognitive functions, especially in the attention/speed domain in the elderly.

## Introduction

Telomeres are nucleoprotein protective caps at the end of the chromosomes containing repeating hexamer, TTAGGG, sequences. They shorten during mitosis due to the inability of the DNA polymerase to complete replication, and due to oxidative stress, they are particularly prone to. During life, there are two phases of accelerated telomere attrition 1) during development up to puberty due to the high number of cell divisions and 2) during aging due to a high level of oxidative stress (1). Telomere shortening destabilizes the genome and leads to the senescence of the affected cells. Cellular senescence is part of the aging process at the organismal level as well (2). In human epidemiological studies, leukocyte telomere length (LTL) is used as a biological marker of aging (3). LTL also reflects the telomere length of other cell types within the body, which is called synchrony. The heritability estimate of LTL is approximately 60%, while that of LTL shortening approximately 30%. Shorter LTL is related to the presence of vascular risk factors such as smoking, obesity, physical inactivity, poor diet, hypertension, and type 2 diabetes mellitus (2, 3). It is associated with age-related chronic vascular and degenerative diseases, especially with Alzheimer's disease and stroke (2, 4). Shorter LTL is also related to all-cause mortality in the elderly (5).

The role of telomeres in normal brain aging is debated. LTL, as well as LTL attrition, has been associated with structural brain changes on magnetic resonance imaging (MRI), including both regional and global brain volumes as well as with white matter hyperintensities (WMH) (6, 7). Results are particularly inconsistent with the effect of short LTL on cognitive function and decline. No relationship was observed in the Dallas Heart Study (DHS) including 2606 subjects between LTL and cognition (8), while recently 2 large meta-analyses studies found an association between longer LTL and higher level of general cognition (9) as well as better cognitive performance and better memory, speed, and executive function (10). Also, longitudinal studies reported associations between LTL attrition and cognitive decline (11).

**Figure 1:**
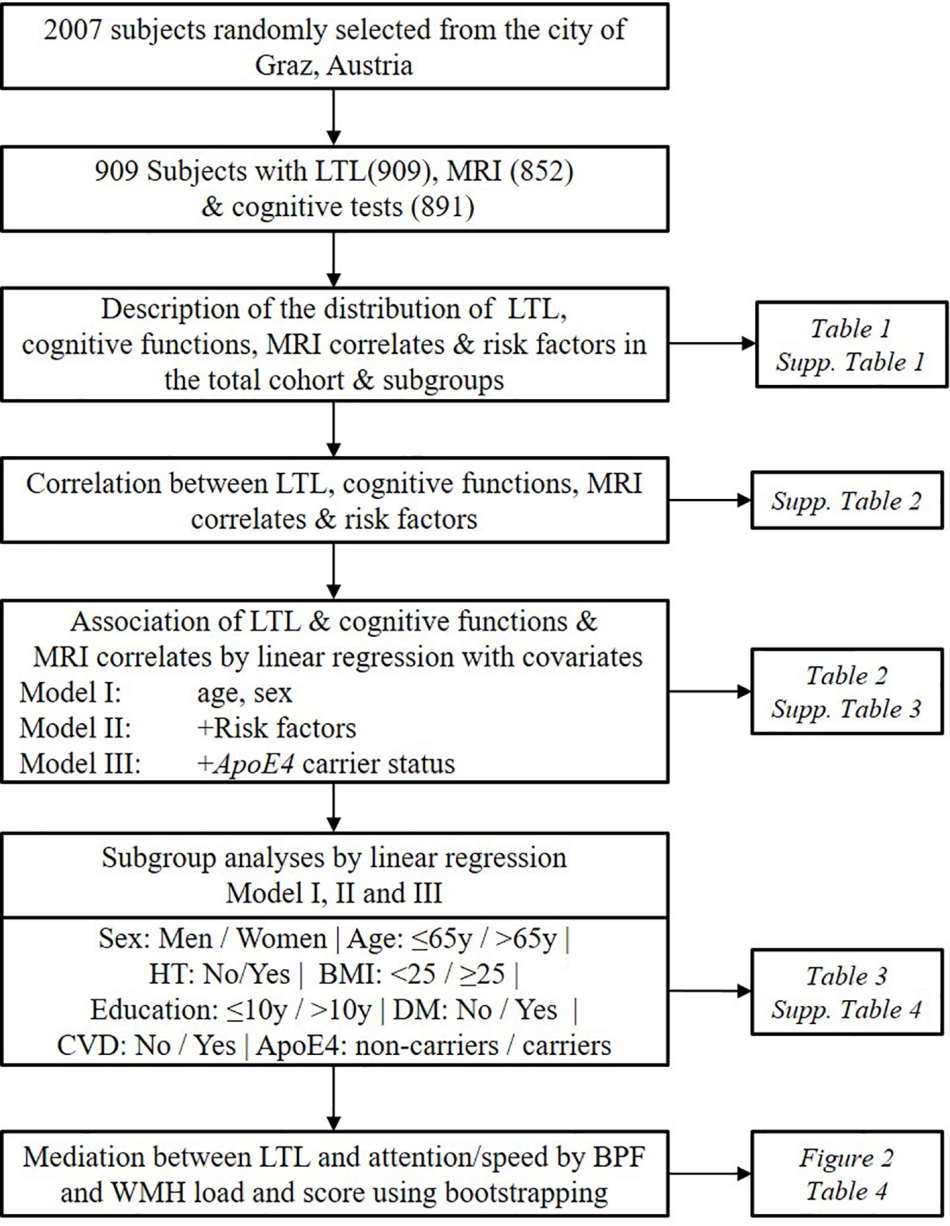
Workflow of the study design. The current study cohort is a subsample of the Austrian Stroke Prevention Study. Risk factors include hypertension, diabetes, cardiovascular diseases, body mass index, education, high-density lipoprotein, and smoking status. LTL, Leukocyte Telomere Length; HT, Hypertension; DM, Diabetes Mellitus; CVD, Cardiovascular Disease; BPF, Brain Parenchymal Fraction; WMH, White Matter Hyperintensities.

Studies investigating the effect of LTL on both brain structure and cognitive function in a simultaneous way at the population level are so far largely missing. Here, we tested the hypothesis that longer LTL is related to 1) better structural preservation of the brain such as larger brain parenchymal fraction (BPF) and less WMH, 2) better cognitive performances including g-factor and composite scores for attention/speed, conceptualization, memory, visuopractical skills. We tested the hypothesis in a large cohort of normal elderly participating in the Austrian Stroke Prevention Study (ASPS) (12). We explored if the effect of LTL on brain phenotypes is modified by the presence of risk factors such as sex, age, hypertension, body mass index (BMI), education, diabetes, cardiovascular disease (CVD), and ApoE4 carrier status using subgroup analyses. We also performed mediation analyses to asses if the observed significant effects of LTL on cognition are mediated by structural brain changes (**Figure 1**). To our knowledge, this is the first study finding evidence that the protective effect of LTL on the brain is highly significant at the structural level and that this, in turn, may transform into better cognition in the attention/speed domain.

## Methods

### Participants

In the present study, we included 909 participants from the ASPS, a community-dwelling cohort study in the elderly population in the city of Graz, Austria, who underwent LTL measurements along with MRI and cognitive tests (12). The study was approved by the Medical Ethics Committee of Karl- Franzens University of Graz. Written informed consent was obtained from all study participants. The mean age of the participants was 65.9 ± 8 years (range: 46–90), 42.7% were males, 69.4% hypertensives, 10.9% diabetics, and 40% had CVD. The mean years of education were 11.3 ± 2.6 (range: 9–18 years). There were 28.3% former smokers and 11% current smokers in the study sample. In total, 898 participants had the ApoE4 genotypes, with 0.8% being homozygous and 19.3% heterozygous carriers.

### LTL Measurement

All 909 participants had LTL measurements. A detailed description of the method was published previously (13). In brief, DNA was extracted from EDTA whole peripheral blood using the phenol-chloroform method. Two quantitative polymerase chain reactions were used to measure telomere repeat copy (T) and single-copy gene (*36B4*) (S). We normalized the relative LTL (T/S ratio) using reference DNA pooled from 24 subjects, and the final T/S ratio was calculated according to Cawthon’s modified method (14). The median of relative LTL was 0.61 (range: 0.05–2.60). The median of LTL was 0.61 (IQR: 0.47 to 0.82).

### Brain MRI

All MRI scans were performed on 1.5T scanners using proton density- and T2-weighted sequences. All punctate early confluent and confluent WMH in the deep and subcortical white matter and periventricular WMHs irregularly extending into the deep white matter were marked and outlined on a transparency that was overlaid on the proton density scan. Periventricular caps, pencil-thin lining, and periventricular halos were not included for WMH load measurement as these changes are considered to be of non-ischemic origin. Independent from visual analysis, WMH load measurements were done on proton density-weighted images on an UltraSPARC workstation (Sun Microsystems, Santa Clara, CA) by a trained operator using DISPImage. The operators used a hardcopy overlaid by the transparency, with each single lesion outlined by the experienced readers as reference. Every single lesion was segmented on the computer image, and its area was provided by the semi-automated thresholding algorithm implemented in DISPImage. Each hyperintensity volume was calculated by multiplying the area by slice thickness. Total WMH load in cubic millimetres was the sum of volumes of single lesions in a given study participant. WMH load was available from 827 participants. WMH score was based on the Fazekas scale on deep white matter changes where a score of 0 is an absence of any white matter change, 1 is punctate foci, 2 is beginning confluence of foci, and 3 is large confluent areas (15). WMH score was available in 852 participants. Brain volume was calculated from the T2-weighted spin-echo sequence using automated structural image evaluation of atrophy^1^. BPF is the ratio of brain parenchymal volume to total intracranial volume (16). BPF was available in 739 subjects.

### Cognitive Testing

For cognitive evaluation, participants went through a group of tests to assess performance in the domains of attention/speed, conceptualization, memory, and visuopractical skills. In detail, information on the conduction of these tests is published previously (12). The measures of the cognitive performance were converted into z-scores by normalizing to the mean of the group. G-factor, which is the first unrotated component of principal component analysis performed on the results of the battery of cognitive tests, was used as a measure of global cognition (17).

### Covariate Analyses

Hypertension was coded as a categorical variable, and a participant considered as hypertensive when there was a history of hypertension or hypertension medication or mean systolic blood pressure ≥ 140 mm of Hg or mean diastolic pressure ≥ 90 mm of hg. Diabetes was defined as a history of diabetes, use of antidiabetic treatment, or fasting blood sugar level >140 mg/dl. Cardiovascular disease assumed to be present if there was evidence of cardiac abnormalities known to be a source for cerebral embolism, evidence of coronary heart disease, appropriate ECG findings, or if an individual presented signs of left ventricular hypertrophy on echocardiogram or ECG (12). Education was measured in years of schooling, meaning the number of years a person attended school including university and higher education programs. Smoking status was assigned after asking each participant whether they ever smoked or if they are currently smoking. It was coded in the form of never, former, or current smoker. Participants were genotyped for the presence of E4 allele using PCR-RFLP and graded as heterozygous (presence of one allele), homozygous (presence of two alleles), or absence of E4 allele (18).

### Statistical Analyses

The statistical analysis was performed using IBM SPSS statistics version 25^2^. The normal distribution of the variables was tested using the Kolmogorov-Smirnov test and by visual inspection of histograms. LTL measurement was transformed into z-scores relative to the mean of the whole group. The z-transformation was done by subtracting the mean LTL of the cohort from the observed value of that individual and dividing by the standard deviation. This was done to interpret the relative position of a particular individual under the LTL distribution curve of the cohort. The skewed distribution of WMH load was converted into normal distribution by log transformation after adding 1 to the volume. BPF was converted into percentage by multiplying the value of fraction by 100 to facilitate the interpretation of the results from linear regression. Cognitive tests had a normal distribution. One outlier with a value of -5.95 within the attention/speed domain was removed.

Co-variates for multiple linear regression models were selected based on their correlation with outcome variables and LTL (Pearson’s correlation p < 0.1) and/or based on previous reports on their association with the phenotypes. Linear regression models were used to test the effect of LTL on brain morphological measures and cognition in the presence of age, sex, risk factors such as hypertension, diabetes, cardiovascular disease, BMI, HDL, years of education, smoking, and ApoE4 genotypes. Model I was adjusted for age and sex, Model II, additionally for hypertension, diabetes, cardiovascular disease, BMI, education, HDL, and smoking status, and Model III for ApoE4 carrier status. We formally tested the collinearity amongst independent variables by calculating variance inflation factors. The variance inflation factor for all independent variables was <1.5, indicating almost no correlation between them.

#### Subgroup Analyses

We divided our cohort into subgroups based on sex (men/women), age (≤ 65y/ > 65y), hypertension (normotensives/hypertensives), BMI (normal weight: < 25 Kg/m^2^/overweight: ≥25 kg/m^2^), education (basic education ≤ 10y/ > 10y mid to high education), diabetes (No/Yes), CVD (No/Yes), and ApoE4 carrier status (No/Yes).

The continuous variables age and BMI were used as covariates in the subgroup analyses within the respective subgroups of age ≤ 65y/ > 65y and, BMI < 25 /BMI ≥25), while years of education was omitted due to only minimal variation left after stratifying on educational status (basic education≤ 10 years subgroup included 250 persons with 9 years and 366 with 10 years of schooling; mid-high education subgroup > 10 years 213 with 13 years and 80 with 18 years of education). We also did not include ApoE4carrier status as covariate in the ApoE stratified analyses for the same reason (7 subjects homozygous, 173 heterozygous for the ApoE4 allele, and 718 non-carriers.)

Due to a large number of statistical tests performed owing to the explorative strategy of this study, we performed the Benjamini-Hochberg procedure to control the false discovery rate (FDR). We used a FDR of 0.05 to calculate the Benjamini-Hochberg critical value and provide the adjusted p-values for each test.

We formally tested if the effect of LTL is modulated by the risk factors by using the interaction terms namely (gender × zLTL), (age × zLTL), (hypertension × zLTL), (BMI × zLTL), (education × zLTL), (DM × zLTL), (CVD × zLTL), and (ApoE4 status × zLTL) in the model III of regression.

### Mediation Analysis

In order to test if the effect of LTL (independent variable) on attention/speed (dependent variable) is mediated either by BPF or WMH, we used bootstrapping (PROCESS macro version 3.4)^3^. We applied model III including age, sex, risk factors such as hypertension, diabetes, cardiovascular disease, BMI, HDL, years of education, smoking, and ApoE4 genotypes as covariates. Bootstrapping gives the estimates of direct and indirect effects. The extent of the effect which was mediated through BPF or WMH was calculated by repeating the sampling procedure 5000 times. Effect sizes, as well as 95% confidence intervals (95%CI), are given. Significant mediation is present when 95%CI does not include the value of zero.

## Results

### Description of the Participants

In total, there were 909 participants with LTL (909), MRI (852), and cognitive tests (891). The mean age was 65.9 years. There were 388(42.7%) men, 631(69.4%) hypertensives, 99(10.9%) diabetics and 364(40%) with CVD within the cohort. The proportion of ApoE4 carriers was 20.1% (**Table 1**, **Supplementary Table 1**). The correlation between LTL and age was negative (r = -0.092, p = 0.006), and participants with CVD had significantly lower LTL (β = -0.136, p = 0.046) than their counterparts (**Supplementary Table 2**). There were no significant differences on LTL by sex, hypertension, BMI, diabetes, education and ApoE4 carrier status (**Supplementary Table 1**). BPF was smaller in individuals with age >65y (p = 2.92 × 10^-42^), hypertension (p = 9.19×10^-7^), DM (p = 7.8 × 10^-4^), and CVD (p = 0.03). WMH load was higher in women (p = 0.002), > 65y (p = 2.7 × 10^-40^), hypertensives (p = 7.2 ×10^-14^), in those with ≤10y of education (p = 0.02), diabetics (p = 0.008), CVD (p = 0.001), and in ApoE4 carriers (p = 0.04). Similarly, WMH score were significantly higher in the subgroups of women (p = 0.03), > 65 y (p = 1.5×10^-32^), hypertensives (p = 2.7×10^-9^), diabetics (p = 0.01), and CVD (p = 0.04) but not in education ≤10y (p = 0.3) and Apoe4 carriers (p = 0.6). The g-factor was significantly lower in all high-risk groups, and also domain specific cognitive scores followed a similar trend. No significant difference was present for conceptualization by sex, for attention/speed and conceptualization by BMI and for visuopractical skills by education (**Supplementary Table 1**).

**Table 1:** Characteristics of the study population.

	Total (n)
**Social demographic factors**	****
Age (years)	65.9 ± 8.0 (909)
Sex (Male)	388 (42.7%)
**Risk factors**	****
Hypertension	631 (69.4%)
SBP (mm of Hg)	143.8 ± 22.8 (908)
DBP (mm of Hg)	87.6 ± 10.0 (908)
Diabetes Mellitus	99 (10.9%)
CVD	364 (40.0%)
BMI (kg/m^2^)	26.9 ± 4.1 (909)
Education	11.1 ± 2.6 (909)
TC (mg/dl)	227.87 ± 40.4 (909)
HDL (mg/dl)	56.5 ± 17.2 (901)
Smoking	
Never	551 (60.7%)
Former	257 (28.3%)
Current	100 (11.0%)
ApoE4	**
No allele	718 (80.0%)
Heterozygous	173 (19.3%)
Homozygous	7 (0.8%)
Predictor	
LTL	0.61(0.47-0.82) (909)
**MRI correlates**	****
BPF (%)	78.7 ± 3.9 (739)
WMH load (mm^3^)	0.8(0.2-3.1) (827)
WMH Score	
0	157 (18.4%)
1	503 (59.0%)
2	123 (14.4%)
3	69 (8.1%)
**Cognitive functions**	****
Attention/Speed	0.03 ± 0.53 (853)
Conceptualization	0.06 ± 0.59 (858)
Memory	0.01 ± 1.01 (860)
Visuopractical Skills	0.00 ± 0.93 (891)
g factor	0.01 ± 1.01 (823)

The independent sample t-test was performed for normally distributed continuous variables, Mann-Whitney U test for skewed variables, and the chi-square test for categorized variables to compare the groups.

For normally distributed variables, mean ± SD, for skewed variables median (IQR), and categorical variables, numbers of observations (%) in the category are given.

SBP, Systolic Blood Pressure; DBP, Diastolic Blood Pressure; CVD, Cardiovascular Diseases; BMI, Body Mass Index; TC, Total Cholesterol; HDL, High-Density Lipoprotein; LTL, Leukocyte Telomere Length; BPF, Brain Parenchymal Fraction; WMH, White Matter Hyperintensities; SD, Standard Deviation; IQR, Inter-Quartile Range.

### LTL Versus MRI Correlates and Cognitive Functions

#### Total Cohort

We found significant correlations between LTL and BPF (r = 0.169, p = 3.71×10^-6^) and attention/speed (r = 0.0081, p = 0.018) (**Supplementary Table 2**). Next, we investigated the association between LTL and MRI as well as cognitive functions by linear regression using 3 models by adjusting for age and sex (Model I), additionally for risk factors such as hypertension, diabetes, CVD, BMI, education, HDL and smoking status (Model II), and adding ApoE4 carrier status (Model III). The results for all analyses in detail are shown in **Supplementary Table 3**. Significant associations were observed between LTL and BPF in Model I (β = 0.44, p = 1.3×10^-4^), II (β = 0.43, p = 2.1×10^-4^), and III (β = 0.43, p = 2.1×10^-4^). The effect of LTL on WMH load as well as the WMH score was significant in Model II (β = 0.02, p = 0.04, β = 0.05, p = 0.04, respectively), and III (β = 0.02, p = 0.04, β = 0.05, p = 0.04 respectively). Results of the regression analyses using Model III for all MRI and cognitive variables are given in **Table 2**. LTL explained 1.2% of the variation in BPF and 0.4% of the variation in WMH load and score. There was no significant association between LTL and any of the cognitive measures in the total cohort in any of the multivariable models (**Table 2** and **Supplementary Table 3**).

**Table 2:** Linear regression analysis of the association of leukocyte telomere length (LTL) with magnetic resonance imaging (MRI) correlates and cognitive functions.

	β	SE	*p*	Partial R^2^
BPF	0.429	0.115	**0.0002**	0.012
WMH load	0.024	0.012	**0.037**	0.004
WMH Score	0.052	0.025	**0.037**	0.004
Attention/Speed	0.025	0.017	0.149	0.002
Conceptualization	0.017	0.019	0.382	0.001
Memory	-0.015	0.029	0.610	0.000
Visuopractical Skills	0.010	0.024	0.668	0.000
g factor	0.009	0.026	0.738	0.000

The results are shown from linear regression after adjusting for age, sex, risk factors, and ApoE4 genotypes (Model III). Bold shows significance level of <0.05.

BPF, Brain Parenchymal Fraction; WMH, White Matter Hyperintensities, β-Effect size, SE-Standard Error.

#### Subgroups

In order to further explore the association, we repeated the linear regression models in subgroups divided by sex, age, hypertension, BMI, education, diabetes, CVD, and by ApoE4 carrier status. Statistically significant results are presented in **Table 3**, while results of the stratified analyses in total are presented in **Supplementary Table 4**. The effect of LTL was highly significant on BPF within the subgroups of women (β = 0.51, p = 0.001), in individuals with age >65y (β = 0.58, p = 0.002), hypertension (β = 0.51, p = 7.0 × 10^-4^), BMI ≥ 25(β = 0.40, p = 0.004), education ≤ 10y (β = 0.42, p = 0.002), CVD (β = 0.33, p = 0.02), in non-diabetics (β = 0.42, p = 4.0 × 10^-4^), and Apoe4 non-carriers (β = 0.49, p = 1.1 × 10^-4^). Although the effect of LTL on BPF in the overweight subjects (BMI ≥ 25) was comparable to that in the normal weight individuals (BMI < 25), in the obese individuals (BMI ≥ 30) the effect of LTL on BPF was three times as high as in non-obese (BMI < 30) (**Supplementary Table 5**). The effect of LTL was significant on both WMH load and WMH score in ≤10y of education, non-diabetics (β = 0.03, p = 0.04, β = 0.03, p = 0.008, β = 0.06, p = 0.02, β = 0.06, p = 0.02), and Apoe4 non-carriers (β = 0.03, p = 0.02, β = 0.07, p = 0.01). On WMH load the effect was in addition significant in hypertensives (β = 0.04, p = 0.02) and on WMH score in subjects without CVD (β = 0.06, p = 0.05). The effect of LTL on cognition was significant only in the attention/speed domain within the subgroup of those with BMI ≥ 25 (β = 0.04, p = 0.05) and ≤10y of education (β = 0.04, p = 0.05). When testing formally for interaction between LTL and the risk factors on MRI and cognitive phenotypes, none of the interaction terms reached statistical significance (p > 0.05).

**Table 3:** Stratified analyses of the significant association of leukocyte telomere length (LTL) with magnetic resonance imaging (MRI) correlates and cognitive functions.

	β	SE	p	Partial R^2^	β	SE	p	Partial R^2^
BPF	Men (313)				Women (426)			
	0.330	0.174	0.058	0.008	0.505	0.155	**0.001**	0.016
	≤65 years (352)				>65 years (387)			
	0.350	0.142	**0.014**	0.013	0.583	0.186	**0.002**	0.016
	Normotensive (231)				Hypertensive (508)			
	0.348	0.187	0.064	0.011	0.505	0.148	0.001	0.015
	BMI <25 (266)				BMI ≥25 (473)			
	0.502	0.202	**0.013**	0.014	0.403	0.140	**0.004**	0.012
	Education ≤10 (505)				Education >10 (234)			
	0.424	0.138	**0.002**	0.012	0.418	0.213	0.051	0.011
	No DM (662)				DM (77)			
	0.418	0.117	**0.000**	0.013	0.250	0.575	0.665	0.002
	No CVD (437)				CVD (302)			
	0.328	0.140	**0.020**	0.008	0.584	0.207	**0.005**	0.017
	Apoe4 non-carriers (589)				Apoe4 carriers (140)			
	0.485	0.125	**0.000**	0.016	0.149	0.299	0.620	0.001
WMH load	Normotensive (258)				Hypertensive (596)			
	-0.003	0.015	0.860	0.000	0.038	0.016	**0.017**	0.008
	Education ≤10 (564)				Education >10 (263)			
	0.029	0.014	**0.039**	0.006	0.018	0.022	0.406	0.002
	No DM (737)				DM (90)			
	0.031	0.012	**0.008**	0.007	-0.076	0.048	0.115	0.021
	Apoe4 non-carriers (589)				Apoe4 carriers (140)			
	0.029	0.013	**0.020**	0.006	-0.001	0.030	0.975	0.000
WMH Score	Education ≤10 (578)				Education >10 (274)			
	0.069	0.030	**0.021**	0.008	0.023	0.047	0.633	0.001
	No DM (760)				DM (92)			
	0.061	0.026	**0.018**	0.006	-0.049	0.102	0.633	0.002
	No CVD (508)				CVD (344)			
	0.060	0.031	**0.049**	0.006	0.042	0.044	0.343	0.002
	Apoe4 non-carriers (674)				Apoe4 carriers (167)			
	0.070	0.027	**0.010**	0.008	-0.019	0.062	0.760	0.000
Attention /Speed	BMI <25 (308)				BMI ≥25 (546)			
	0.001	0.034	0.974	0.000	0.039	0.019	**0.048**	0.006
	Education ≤10 (581)				Education >10 (273)			
	0.044	0.022	**0.048**	0.005	-0.031	0.025	0.208	0.005

The results are shown from linear regression after adjusting for age, sex, risk factors, and ApoE4 genotypes (Model III) in subgroups. Bold shows significance level of <0.05. For each subgroup, number of participants is given in the brackets.

BPF, Brain Parenchymal Fraction ;WMH, White Matter Hyperintensities; BMI, Body Mass Index; DM, Diabetes Mellitus; CVD, Cardiovascular Disease, β-Effect size, SE-Standard Error.

By controlling for multiple testing using FDR, the significant association between LTL and BPF within subgroups of women (p = 0.005), individuals with age >65y (p = 0.006), hypertension (p = 0.004), BMI ≥ 25 (p = 0.009), education ≤ 10y (p = 0.006), CVD (p = 0.010), in non-diabetics (p = 0.003), and Apoe4 non-carriers (0.002) persisted. Yet, the associations between LTL and WMH load, WMH score and attention/speed domains within respective subgroups became non-significant (p > 0.05).

**Figure 2:**
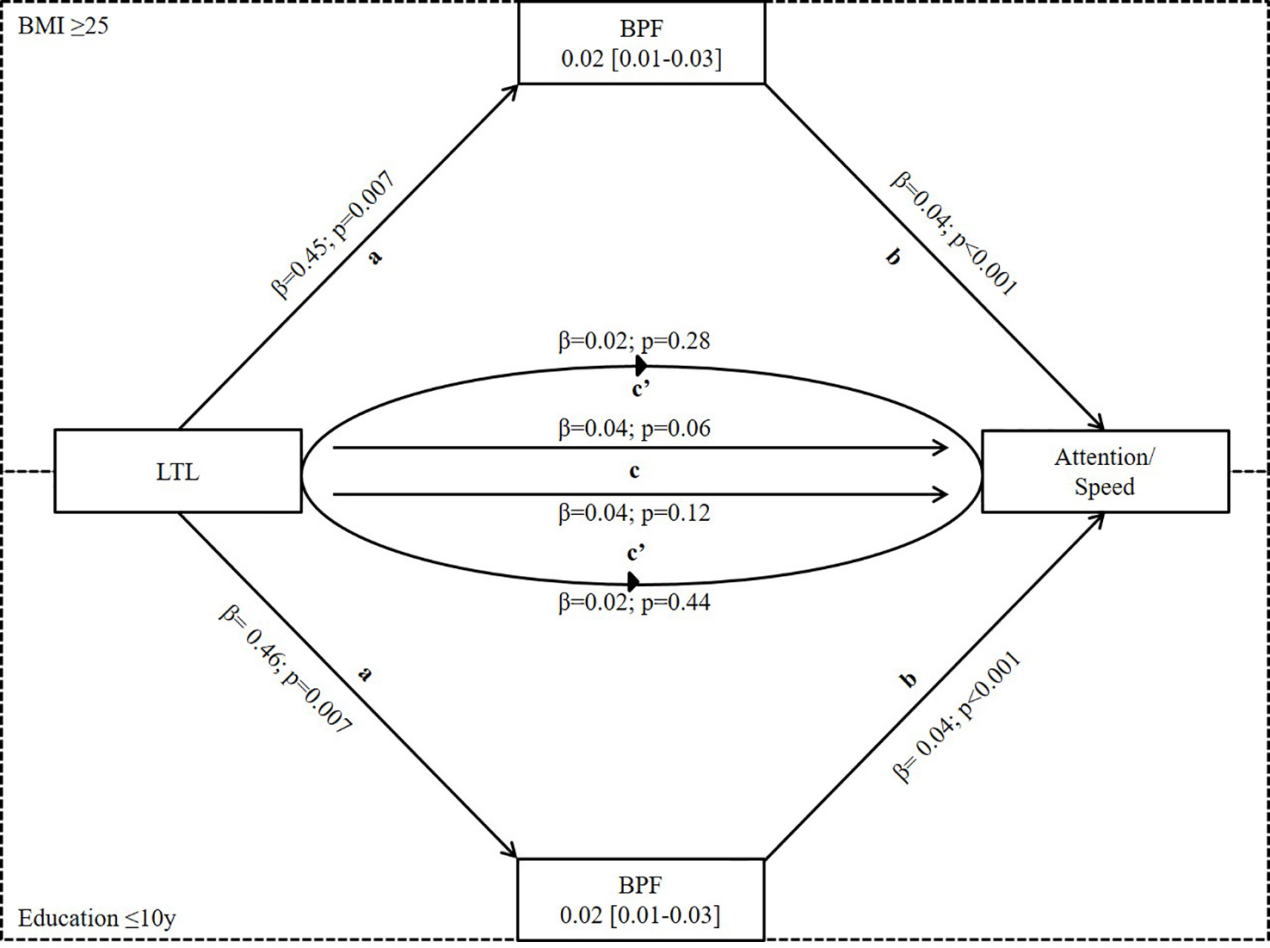
Mediation analysis of LTL on attention/speed *via* BPF in subgroups of BMI≥ 25 and Education ≤ 10y where LTL was significantly associated with both BPF and attention/speed. Mediation is calculated after adjusting for covariates from Model III of linear regression. a- effect of LTL on BPF, b-effect of BPF on attention/speed, c-total effect of LTL on attention/speed, and c’-direct effect of LTL on attention/speed when controlled for BPF. It shows partial mediation because total effect (c) of LTL on attention/speed is reduced in the presence of BPF (c’). LTL, Leukocyte Telomere Length; BPF, Brain Parenchymal Fraction.

Using bootstrapping, we investigated if the significant effects of LTL on attention/speed were mediated by BPF in the subgroup of BMI ≥ 25 and education ≤10y or by WMH load/score in those ≤10y of education using model III. We found significant mediation by BPF in both subgroups (effect = 0.02, 95%CI 0.01-0.03 for both subgroups) while the mediation effect by WMH load/score was not significant (load: effect = -0.01, 95%CI -0.02-0.001; score: effect = -0.03, 95%CI -0.01-0.001) (**Figure 2** and **Table 4**).

**Table 4:** Mediation analyses.

Subgroups	Effect (β)	SE	CI Lower	CI Upper
BPF				
BMI ≥25	0.019	0.006	0.007	0.033
Education ≤10	0.019	0.006	0.008	0.032
WMH load				
Education ≤ 10	-0.008	0.005	-0.019	0.001
WMH Score				
Education ≤ 10	-0.0029	0.0026	-0.0088	0.0013

The effect size of the mediation calculated using bootstrapping is shown here. We use the 5000 bootstrap samples. When the confidence interval of the effect size does not include zero, it shows a significant effect.

BPF, Brain Parenchymal Fraction; WMH, White Matter Hyperintensities; BMI, Body Mass Index; SE, Standard Error; CI, 95% Confidence Interval.

## Discussion

### Summary

In summary, we found a highly significant association between LTL and BPF in the elderly. The association was independent of age, sex, the presence of vascular risk factors, and ApoE4 allele. The effect was stronger in women and in individuals with a higher risk for cerebrovascular disease and dementia such as those older than 65 years, hypertensives, overweight (BMI ≥ 25), having education less than 10 years, and CVD. In the case of ApoE4, the effect of LTL on BPF was significantly stronger in non-carriers. LTL had no significant effect on cognition except for the domain of attention/speed within the subgroups of basic education and overweight. The association between LTL and attention/speed was significantly mediated by BPF in these subgroups.

### LTL and MRI Correlates

There are only a few studies available, which investigated brain size in relation to LTL. Their results are contradictory. The so-far largest study, the DHS with 1960 subjects including non-Hispanic whites, blacks, Hispanics, and other ethnicities, found a highly significant association with total cerebral volume and volume of the cerebral white and grey matter (6). LTL explained 1.3% of the cerebral volume, which is very close to that, what we observed for LTL in the case of BPF (partial R^2^ = 1.2%) (**Table 2**). In both studies, this corresponded about 1/20 of the proportion explained by age (partial R^2^ = 20 in DHS and R^2^ = 24 in ASPS). This pinpoints to a robust and reproducible effect of LTL on brain size, even in diverse populations. In our cohort, the effect of LTL on BPF was especially strong in the high-risk groups. The effect size of LTL in women, individuals older than 65 years, hypertensives, and those with cardiovascular disease was approximately twice as large as in their counterparts. The highest protective effect of LTL was seen in the subgroups of obese people where the effect size was three times as high as in their counterpart (BMI < 30). These results suggest an especially important protective role for longer telomeres when the brain is exposed to damaging factors. Similar subgroup-specific results were reported by the Swedish subsample of the CASCADE where the effect of LTL on subcortical atrophy was stronger in those above the median age of 69.6 years (19). Importantly, in the case of ApoE, those who did not carry the E4 allele, the effect of LTL on BPF was more than three times as high (p = 0.0001) as in carriers (p = 0.6). This was a surprising finding and might indicate a different mechanism linking LTL to BPF in this subgroup as in the traditional high-risk groups.

For both WMH load and score, we observed an unexpected positive association with LTL, meaning longer telomeres were related to more lesions in the total cohort as well as in some subgroups (**Supplementary Tables 3** and **4**). The significance and the effect sizes were moderate but became more significant and, larger when adjusting for vascular risk factors and ApoE4 carrier status in addition to age and sex. In contrary, a recent study (7) on 369 American Indians found a significant association between shorter LTL and an increase in WMH loads. Also, the Swedish subsample of CASCADE reported shorter LTL being associated with periventricular WMH particularly in participants above the age of 69.6 years (19), We did not see the differential effect by age, rather was the association significant within hypertensives, non-diabetics, and those with education less than 10 years. Most likely, the differences between the subgroups were at least partly due to differences in sample sizes. Relevantly different effect sizes were only observed for WMH load in hypertensives vs normotensives and ApoE4 non-carriers vs carriers. Presently we have no biological explanation for the detrimental effect of longer telomeres on WMH load and score. They may represent chance findings, as after performing FDR for adjusting for multiple comparisons, the significance does not persist.

### LTL and Cognition

In our study, we applied a comprehensive evaluation of cognitive functions within the domains of attention/speed, conceptualization, memory, and visuopractical skills, and built the composite score of g-factor as a measure of global cognitive function. LTL had a very specific effect on cognition with longer telomeres being associated with better performances only in the attention/speed domain. However, this effect was only observed in those with overweight and education less than 10 years. In these groups, the effect sizes were also much larger than in their counterparts. Previously population-based cohort studies reported divergent findings. While in DHS, there was no association observed within 2,606 participants between LTL and cognition (8), Zhan et al. reported that longer LTL was related to better general cognition in a meta-analyses including four prospective cohorts (N = 5,955) when adjusted for age. This association became insignificant when additional adjustment for risk factors was done. In the largest meta-analyses so far performed in European ancestry cohorts (N = 17,052) (10) longer LTL was associated with better cognitive performance, including memory, executive function, and importantly similarly to our study with speed.

### Effect Mediation by BPF on Attention/Speed

The effect of LTL was significant on both BPF and attention/speed in subjects with overweight and education less than 10 years. Within these subgroups, we found evidence that about half of the effect by LTL on cognition was mediated by BPF. Our results for the first time support the hypothesis that longer telomeres protect the brain, and this transforms to better cognitive performance especially within the attention/speed domain. Attention/speed is considered as a basic cognitive function, which declines with age linearly, and its decline also affects other cognitive domains (20, 21). In our cohort, BPF was only significantly related to attention/speed but not to other cognitive domains or to g-factor when tested by linear regression using model III.

### Working Hypothesis

**Figure 3:**
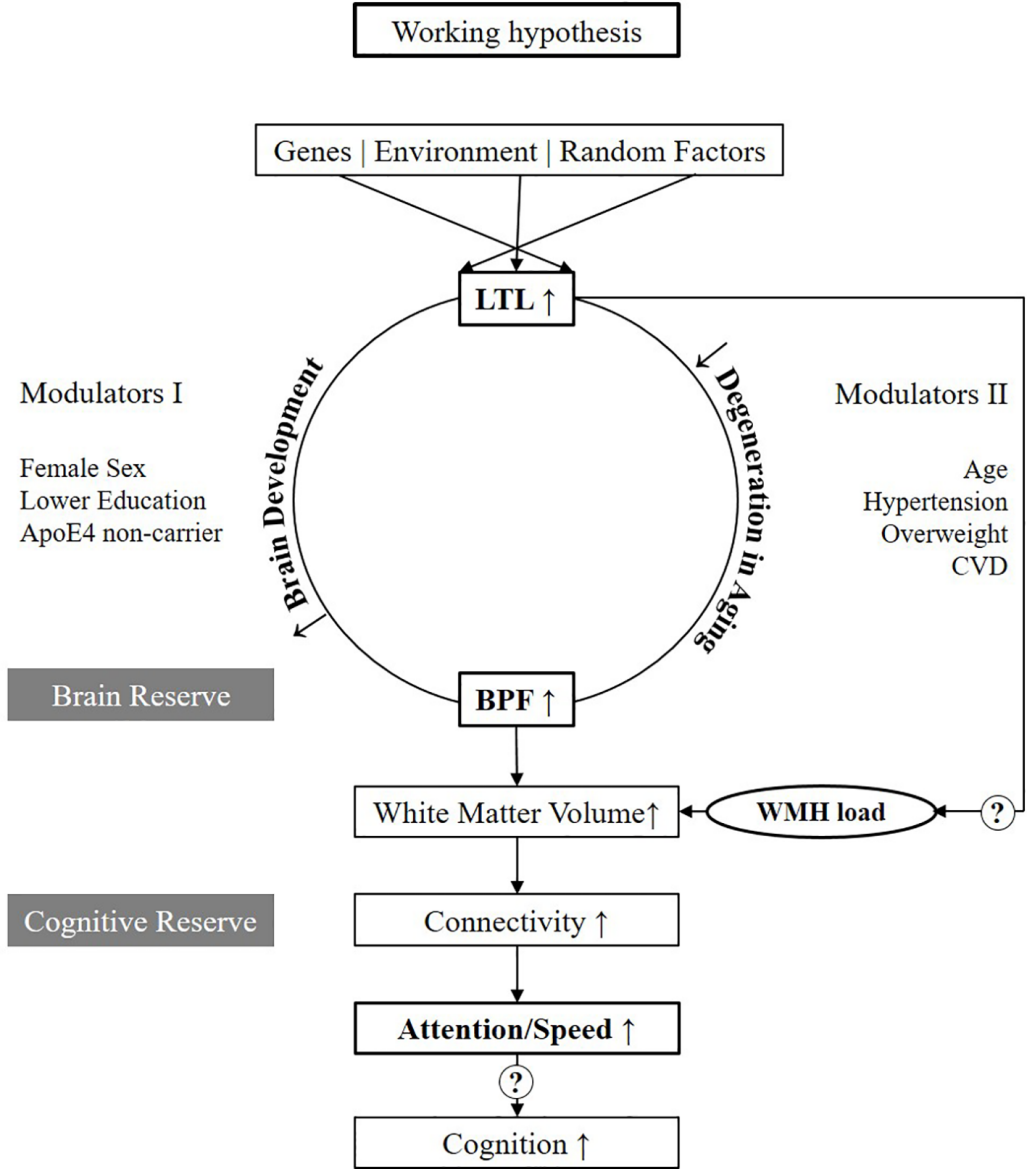
Working hypothesis. Variables in bold are those which are tested in the present study. LTL, Leukocyte Telomere Length; BPF, Brain Parenchymal Fraction; WMH, White Matter Hyperintensities; CVD, Cardiovascular disease.

**Figure 3** presents our working hypothesis on the relation between LTL and structural and functional brain changes associated with aging. In this model, we hypothesize that longer LTL plays a causal and protective role in the process of brain aging. Mendelian randomization studies suggested a causal role for telomeres for Alzheimer's Disease (22, 23). Its effect on BPF might be mediated by two major pathways, one linked to the development of the brain up to puberty and the second related to aging. These are the two phases when telomere attrition is accelerated, and major inter-individual differences in telomere length develop (3). In the first pathway, we hypothesize that those inheriting longer telomeres or loosing less of their telomere during growth, develop a larger brain translating to higher brain reserve. Indeed, faster LTL attrition in younger life was associated with poorer global cognitive function as well as worse performance in domains including processing speed in midlife (24). We further hypothesize that the effect modulators acting already early in life such as female sex, lower education, and ApoE4 non-carrier status, mainly act on this developmental pathway (Modulators I). In the second pathway, we hypothesize that in those with longer LTL, the process of brain aging is slowed down, and brain parenchyma is longer preserved. In addition, during aging, the brain is challenged by risk factors such as old age, hypertension, overweight and CVD (Modulators II). Those, however, who enter the process of aging with a larger brain reserve are better protected against atrophy and cognitive decline. This is in line with the findings of Brickman et al. (2011), showing that for any given level of cognitive function, those with higher reserve -derived by latent variable analyses have higher WMH load (25). A difference, which especially gets measurable when risk factors are present. Our data presently only supports the connection between longer LTL, larger BPF, and better performances in attention/speed (marked bold in **Figure 3**). However, it will be important to further investigate in larger studies if better attention/speed further translates to better cognitive functions in other domains as well. Also, the microstructural substrate of the hypothesized larger brain reserve by more advanced MRI such as DTI needs to be followed up (26). The peak width of skeletonized mean diffusivity was reported to be strongly linked to attention/speed (27). Clearly, also, the association between LTL and WMH load needs further investigation.

### Strengths and Weaknesses

The strength of our study is the large sample size within a well-established population-based cohort with LTL, MRI, and comprehensive measures on cognitive functions. This also allowed us to explore the effect of LTL on the brain by stratifying on subgroups based on the presence and absence of relevant risk factors and define significant effect modulators. Yet, in some subgroups such as those with DM, the sample size was still probably too small to reach statistical power for meaningful testing. By performing mediation analyses between LTL, BPF and cognition, we found the first evidence that the beneficial effect of LTL on BPF transforms into a positive effect on attention/speed. Certainly, using this explorative strategy, we performed multiple tests. Altogether we ran the 3 models within 16 subgroups for all MRI and cognitive phenotypes. Indeed, when adjusting for multiple testing by using FDR, significant results were only present for BPF (p < 0.05) but not for WMH (p = 0.1) or attention/speed (p = 0.2). A weakness of the study is its cross-sectional setting, which makes causal interference difficult as well as the possibility for residual confounding in spite to test for a wide range of possible confounders. Since this study was based on the original ASPS cohort, only 1.5T MRI was available. In the future, we, therefore, will expand our study to the ASPS-Family cohort with available 3T MRI scans in order to deepen our understanding of microstructural changes involved in the brain effect of LTL.

## Conclusion

Our results support a significant protective role of longer LTL on the brain in the normal elderly. Longer LTL is associated with a larger brain and with better cognitive functioning in the attention/speed domain. The effect is largely confined to women and to the high-risk groups of older than 65 years, overweight, hypertensives and those having education less than 10 years. We found for the first time evidence that the protective effect of LTL on the brain may transform into better cognition as up to 50% of its effect on attention/speed was mediated by BPF. Our results pinpoint to a possible role of longer LTL in the maintenance of better brain and cognitive reserve in the elderly, which might be especially important when the brain is challenged by risk factors.

## Data Availability Statement

The datasets generated for this study are available on request to the corresponding author.

## Ethics Statement

The studies involving human participants were reviewed and approved by the ethics committee of Medical University of Graz. The patients/participants provided their written informed consent to participate in this study.

## Author Contributions

PG—Data analyses and writing the manuscript. RS—Critically reading the manuscript and principal investigator of ASPS study. HS—Study design, data analyses, writing the manuscript and obtained funding for the project.

## Funding

The research reported in this article was funded by the Austrian National Bank Anniversary Fund, P15435, City Graz, Graz, Austria and, the Austrian Ministry of Science under the aegis of the EU Joint Programme—Neurodegenerative Disease Research—www. jpnd.eu. The project is supported through the following funding organizations under the aegis of EU Joint Programme—Neurodegenerative Disease Research—www.jpnd.eu: Australia, National Health and Medical Research Council, Austria, Federal Ministry of Science, Research and Economy; Canada, Canadian Institutes of Health Research; France, French National Research Agency; Germany, Federal Ministry of Education and Research; Netherlands, The Netherlands Organisation for Health Research and Development; United Kingdom, Medical Research Council. This project has received funding from the European Union’s Horizon 2020 research and innovation program under grant agreement no. 643417. PhD position for PG is supported through the PhD program “Molecular Medicine” of Medical University of Graz, Graz, Austria.

## Conflict of Interest

The authors declare that the research was conducted in the absence of any commercial or financial relationships that could be construed as a potential conflict of interest.

## Footnotes

1 SIENEX, part of FMRIB Software Library; https://fsl.fmrib.ox.ac.uk/fsl/fslwiki/

2 IBM Corp. Released 2017. IBM SPSS Statistics for Windows, Version 25.0. Armonk, NY: IBM Corp.

3 The process macro for SPSS and SAS; http://processmacro.org/index.html
